# Linking Swedish health data registers to establish a research database and a shared decision-making tool in hip replacement

**DOI:** 10.1186/s12891-016-1262-x

**Published:** 2016-10-04

**Authors:** Peter Cnudde, Ola Rolfson, Szilard Nemes, Johan Kärrholm, Clas Rehnberg, Cecilia Rogmark, John Timperley, Göran Garellick

**Affiliations:** 1 Swedish Hip Arthroplasty Register, Centre of Registers Västra Götaland, Medicinargatan 18G, SE 413 45 Gothenburg, Sweden; 2 Department of Orthopaedics, Institute of Clinical Sciences, Sahlgrenska Academy, University of Gothenburg, Gothenburg, SE 413 45 Sweden; 3 Medical Management Centre, Karolinska Institutet, Tomtebodavägen 18a, Solna, Sweden; 4 Department of Orthopaedics, Lund University, Malmö University Hospital, SE-205 02 Malmö, Sweden; 5 Department of Orthopaedics, Hywel Dda University Healthboard, Prince Philip Hospital, Bryngwynmawr, Llanelli, SA14 8QF UK; 6 Hip Unit, Princess Elizabeth Orthopaedic Centre, Royal Devon & Exeter Hospital Barrack Road, Exeter, EX2 5DW UK

**Keywords:** Database, Socio-economic, Comorbidities, Total Hip Arthroplasty, Revision, PROM, Shared Decision Model

## Abstract

**Background:**

Sweden offers a unique opportunity to researchers to construct comprehensive databases that encompass a wide variety of healthcare related data. Statistics Sweden and the National Board of Health and Welfare collect individual level data for all Swedish residents that ranges from medical diagnoses to socioeconomic information. In addition to the information collected by governmental agencies the medical profession has initiated nationwide Quality Registers that collect data on specific diagnoses and interventions. The Quality Registers analyze activity within healthcare institutions, with the aims of improving clinical care and fostering clinical research.

**Main body:**

The Swedish Hip Arthroplasty Register (SHAR) has been collecting data since 1979. Joint replacement in general and hip replacement in particular is considered a success story with low mortality and complication rate. It is credited to the pioneering work of the SHAR that the revision rate following hip replacement surgery in Sweden is amongst the lowest in the world. This has been accomplished by the diligent follow-up of patients with feedback of outcomes to the providers of the healthcare along with post market surveillance of individual implant performance. During its existence SHAR has experienced a constant organic growth. One major development was the introduction of the Patient Reported Outcome Measures program, giving a voice to the patients in healthcare performance evaluation. The next aim for SHAR is to integrate patients’ wishes and expectations with the surgeons’ expertise in the form of a Shared Decision-Making (SDM) instrument. The first step in building such an instrument is to assemble the necessary data. This involves linking the SHARs database with the two aforementioned governmental agencies. The linkage is done by the 10-digit personal identity number assigned at birth (or immigration) for every Swedish resident. The anonymized data is stored on encrypted serves and can only be accessed after double identification.

**Conclusion:**

This data will serve as starting point for several research projects and clinical improvement work.

## Background

Involving patients and discussing risks and expected benefits of medical interventions is an integral component of the shared decision-making (SDM) process [1]. Although widely endorsed, the implementation of SDM in clinical practice remains limited, partly due to the lack of tools to display evidence and to support the process [2, 3]. One of the main challenges in constructing such a SDM tool is to gather sufficient and relevant data. As data often needs to be collected from different sources and come in different shapes and formats, the limiting factor is not the scale but the heterogeneity of the data [4]. In order to succeed with SDM tools, fine-grained data are required to build models relevant and valid at individual level [5]. This immediately highlights a common hurdle that many researchers face whereby data is stored by different organizations using a variety of indexing systems making linkage-studies near impossible. Sweden facilitates linkage-studies by the adoption of the 10-digit personal identity number (PIN) maintained by the Swedish Tax Agency. Use of this 10-digit PIN is not without problems but it allows a 100 % coverage of the Swedish healthcare system and is instrumental for linkage between population and health data registers [6]. Alongside with the top-down initiated governmental agencies, an increasing number of nationwide Swedish Healthcare Quality Registers (QRs) focusing on specific disorders have been initiated during the past two decades [7]. Linkage of the QRs with data from the governmental agencies provides relevant supplemental data not recorded by the QRs. Analysis of this powerful combined data highlights the need for adequate measures to preserve confidentiality [8].

**Fig. 1:**
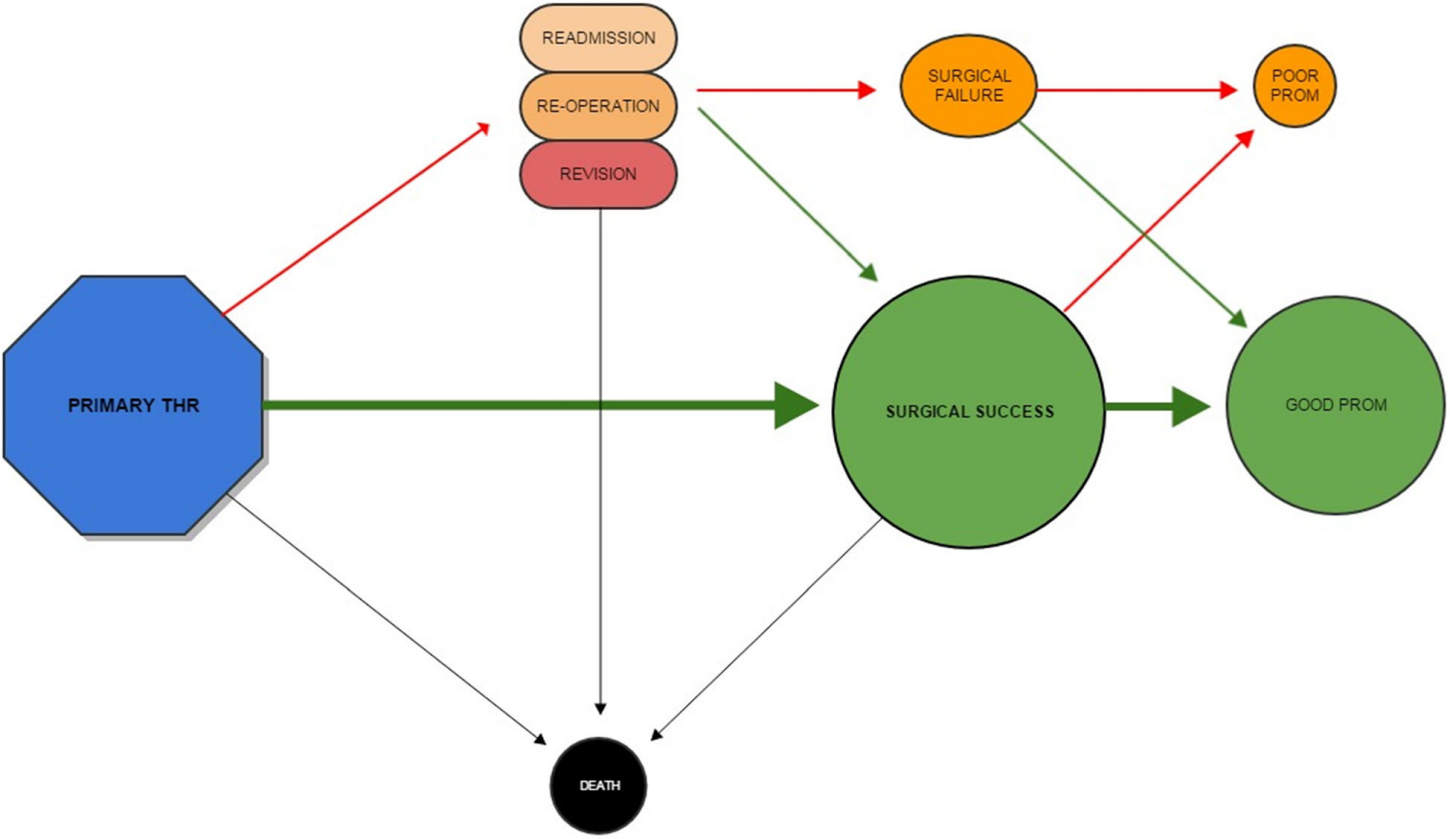
Patient pathway

Hip and knee osteoarthritis are major contributors to global disability [9]. Joint replacement is generally considered a successful treatment to decrease pain and to improve function and health-related quality of life (HRQoL) when non-surgical treatment options do not suffice [10]. Advances in surgical techniques, implants, and perioperative care over the last decades have contributed to improved outcomes following joint replacement at group level. SHAR has been instrumental in identifying some implants that have not performed well such that they have been removed from the market. Overall, however, total hip replacement has been recognized as achieving outstanding outcomes for patients and has been called the “operation of the (20^th^) century” [11]. There remain concerns about the ability to predict outcomes at individual level and the increasing number of joint replacements [12, 13] will create an even greater financial burden for health care systems if treatment is not optimized for all cases. Furthermore, there is no consistent definition of what can be considered a successful joint replacement and outcome measures are not harmonized. A multidimensional outcomes assessment not only includes mortality, reoperation frequency and other adverse events but also pain reduction, functional improvement and patient satisfaction (Fig. 1.)

The decision to proceed with joint replacement surgery should be based on patient preferences, and the balance between benefits conferred and the risk of complications [14]. It is essential to consider patient values and preferences along with the physicians’ experience and expertise [15].

The aim of the present paper is to describe the steps taken by the Swedish Hip Arthroplasty Register (SHAR) in acquiring data that would facilitate the construction of a SDM tool for patients considering hip replacement. We describe the sources from which data have been retained and the research projects that are planned.

## Construction and content

**Fig. 2:**
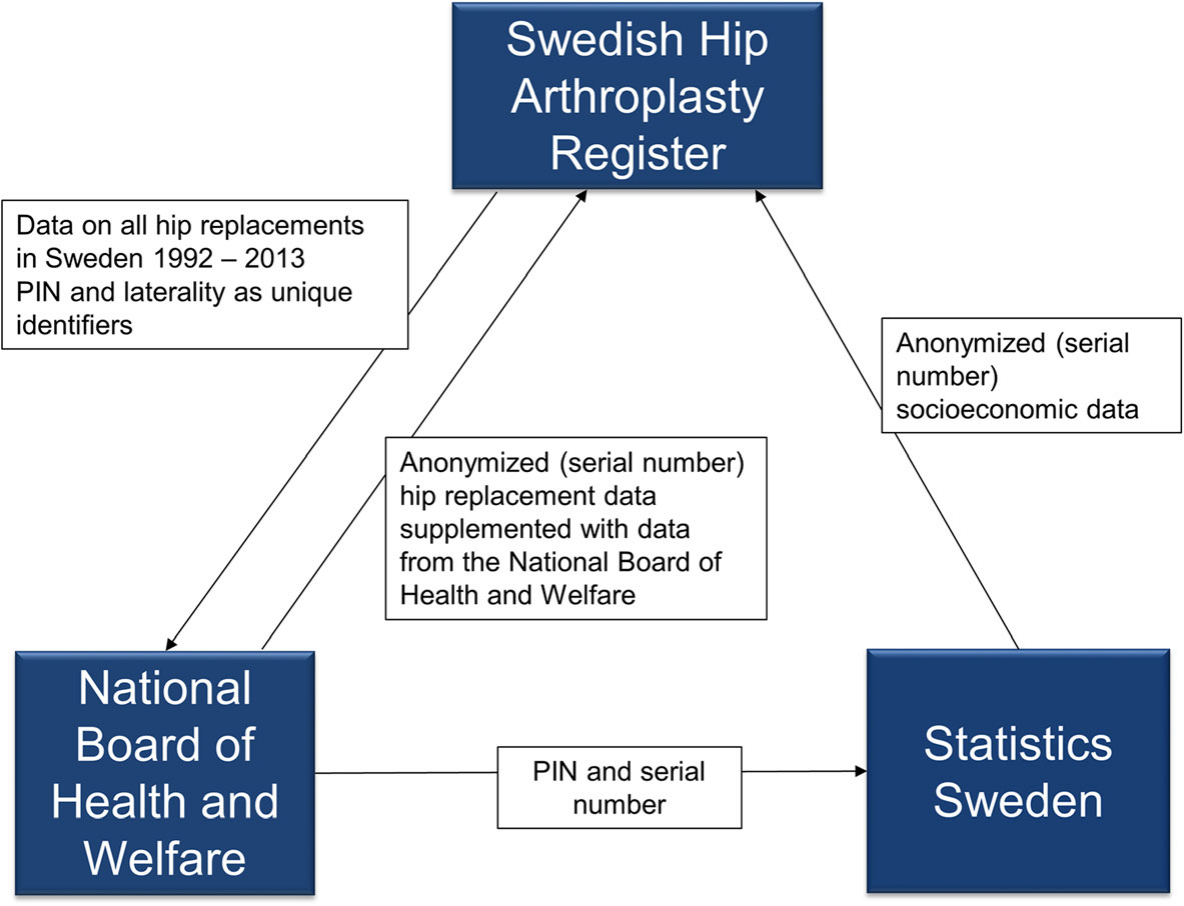
Data sources and workflow

Data have been acquired from three main sources. The starting point was the SHAR (Fig. 2). After ethical approval from the Regional Ethical Review Board in Gothenburg (dnr 271-14) we identified all first time (primary) hip replacement surgeries in Sweden from 1992 to 2013 using SHAR data. For each primary hip replacement, data was organized by PIN and laterality (left or right hip) and all available SHAR data related to the primary intervention (Table 1) was added. This data set was submitted to the National Board of Health and Welfare. Using the PIN as the unique identifier, the National Board of Health and Welfare added the requested variables (Table 1). At this stage the PIN was removed and replaced with a serial number in order to anonymize the data before the data set was transmitted to SHAR. The key for linking the serial numbers to the PIN is saved at the National Board of Health and Welfare and researchers will under no circumstances be granted access to this key. Next, the National Board of Health and Welfare forwarded the list of PINs and serial numbers directly to Statistics Sweden to obtain requested socioeconomic variables (Table 1). Finally, Statistics Sweden returned a data set to SHAR with the serial number and laterality as the unique identifiers. In the following we give a short description of each data source.

**Table 1:** Overview of the data from the Swedish Hip Arthroplasty Register, Statistics Sweden and the National Board of Health and Welfare

Swedish Hip Arthroplasty Register		Statistics Sweden		National Board of Health and Welfare	
Variable Category	Variable	Variable Category	Variable	Variable Category	Variable
*Patient factors*		Demography	Place of birth	*National Patient Register*	
Demography	Year of birth		Residency	Diagnosis	International classification of diagnosis (ICD-9/ICD-10)
	Sex		Relocation within Sweden, emigration		Diagnosis related grouping (DRG)
	Weight		Marital status		Main diagnose
	Height		Socioeconomic classification (Care Need Index)	Interventions	Classification of interventions (KVÅ)
Diagnosis (at hip)	International classification of diagnosis (ICD-10)	Income	Individual disposable income	Date	Admission
	Laterality		Family disposable income		Discharge
	American Society of Anaesthesiologists (ASA) Physical Status classification	Family circumstances	Family type		Elective or acute care
*Surgical and implant-related variables*			Children at home	Hospital	Hospital name
Hospital	Hospital identifier		Number of people in the household		Clinic
Date	Date of surgery	Education	Educational attainment		Administrative category (rural, central, university, private)
Type of surgery	Primary, revision, reoperation		Year of graduation from highest education	Type of care	Outpatient/inpatient
	Total, partial, resurfacing hip replacement				
Implants characteristics	Manufacturer	Sickness benefits	Days with sickness compensation	*Cause of Death Register*	
	Model		Total income due to sickness	Cause of death	International classification of diagnosis (ICD-9/ICD-10)
	Size		Days with occupational injury compensation	Date	Date of death
	Type of implant fixation		Total income due to occupational injury	*Drug Register*	
Surgery details	Surgical approach		Days with rehabilitation compensation	Date	Prescription date
*Patient-reported variables (preop and 1, 6, 10 years postop)*			Total income due to rehabilitation		Withdrawal date
Date	Date of completion	Unemployment benefits	Unemployment classification	Drug	Name
Musculoskeletal comorbidity	Charnley class		Total income from unemployment compensation		Dosage
Pain	Visual analogue scale (VAS) hip pain		Days unemployed		Anatomic Therapeutic Chemical classification system (ATC)
Health-related quality of life	EuroQol 5 dimensions		Days partly unemployed	Cost	Patient cost
	EuroQol VAS	Welfare benefits	Family welfare benefits		Subsidized cost
Satisfaction	Satisfaction VAS		Housing benefits	*Cancer Register*	
Treatment prior to THR	Physiotherapy			Diagnosis	International classification of diagnosis (ICD-7 up to ICD-10)
	Patient education				Type (malignant, non-malignant)
					Localisation/laterality
					Metastasis
					Morphologic classification
					Date of diagnosis

### Swedish hip arthroplasty register

**Fig. 3:**
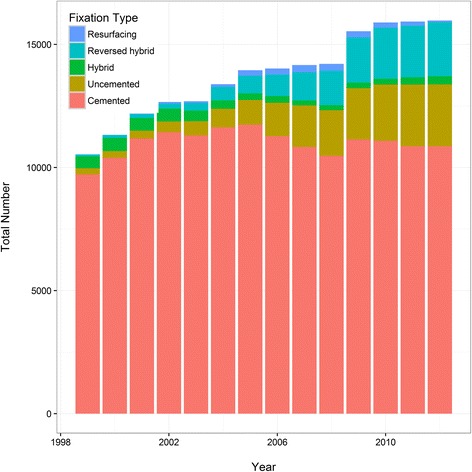
Number of total hip replacements in Sweden from 1999 through 2012 and fixation type

**Fig. 4:**
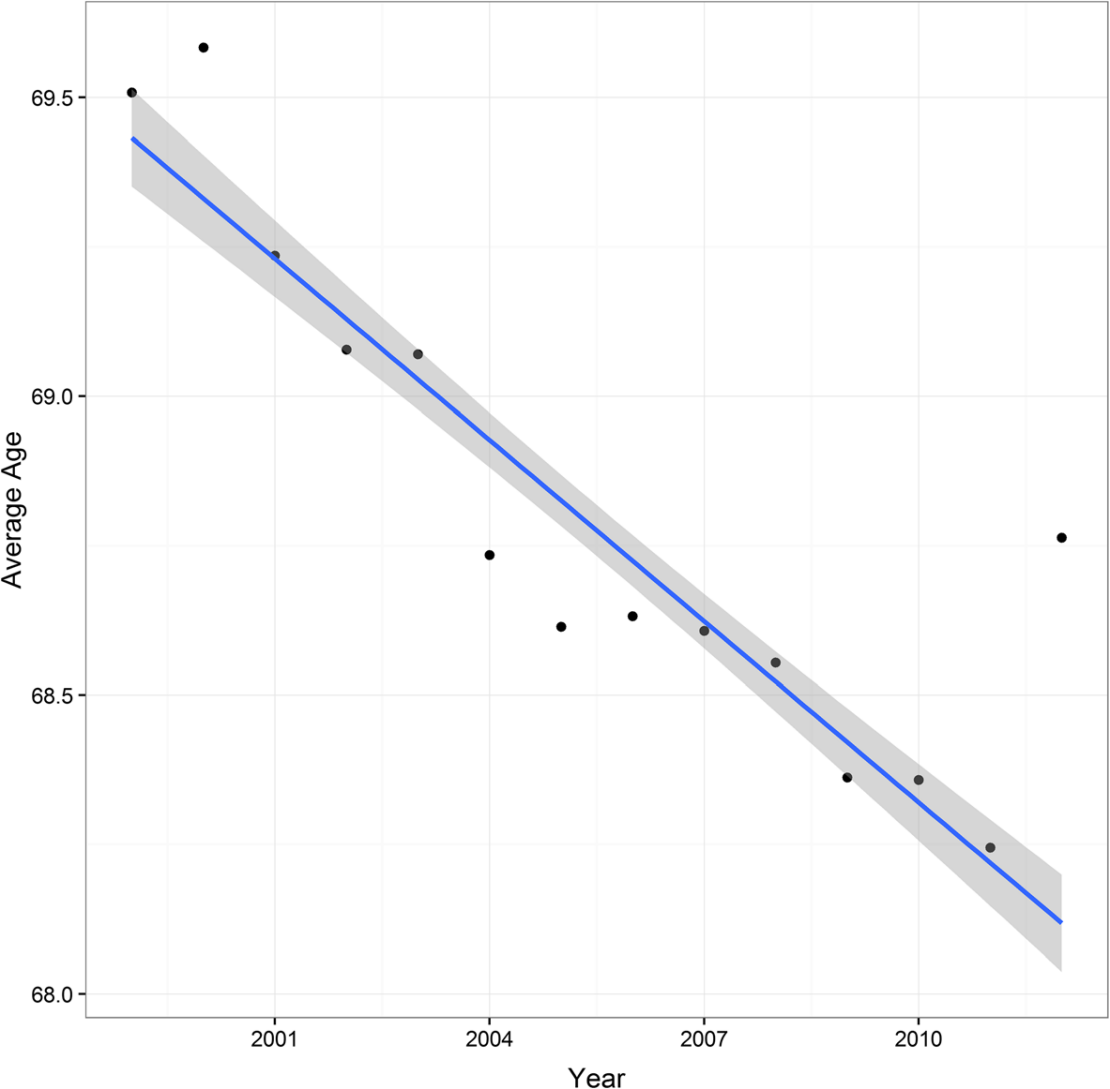
Average distribution of age at time of surgery in patients undergoing total hip replacements in Sweden from 1999 through 2012 with the estimated trend line (*blue line*) and associated 95 % pointwise confidence band (*grey shaded area*)

**Fig. 5:**
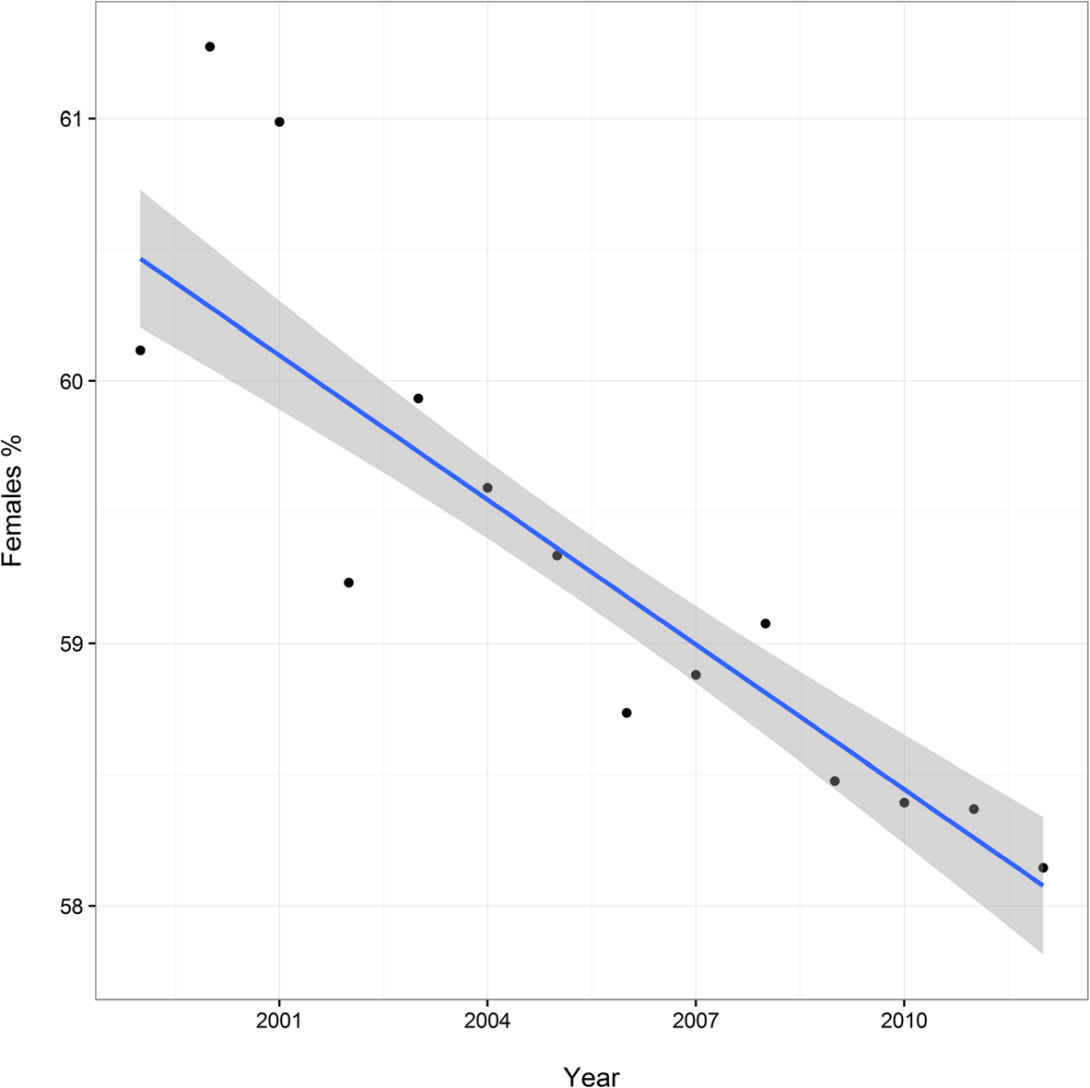
Gender distribution in patients undergoing total hip replacement in Sweden from 1999 through 2012 with the estimated trend line (*blue line*) and associated 95 % pointwise confidence band (*grey shaded area*)

SHAR prospectively collects data on all hip replacement surgeries. The Register started its activities in 1979 and is one of the oldest QRs in Sweden. Before 1992, each participating hospital submitted aggregated data on primary THR’s. Since 1992, all hospitals report primary surgeries and subsequent operations based on the unique PIN and laterality. Since 1992 there has been a progressive increase in primary procedures recorded in the SHAR with a decreasing revision burden. Some examples of changes in volume, fixation type and demography can be found in Figs. 3, 4 and 5 (Figs. 3, 4 and 5). The data collection was expanded to also include partial hip replacement (PHR, also referred to as hemi-arthroplasty in the literature) and this has been recorded since 2005. The Register continuously validates data quality and the annual assessment of completeness (number of reported hip replacements/number of performed hip replacements) has been 98–99 %, even though it is not mandatory for the clinics to report to SHAR. An thorough description of the chronology of the development and the growth of the SHAR has been provided previously [16].

SHAR have three main tasks related to hip replacement surgery: analysis of healthcare institutions and their activities, to stimulate continuous clinical improvement and to perform clinical research. Additionally SHAR manages post-market surveillance of implants.

For the data set and proposed projects outlined here, SHAR contributes with data on patient demography, implant related technical details, surgical details and patient-reported outcomes (Table 1). The latter is collected through the Patient-Reported Outcome Measure (PROM) program which was started in 2002 and attained full coverage in 2008 [10]. Within the PROM program, all elective patients are invited to complete a short questionnaire preoperatively and at 1, 6, and 10 years postoperatively.

### The National Board of Health and Welfare

The National Board of Health and Welfare is the main administrative authority of the Swedish healthcare system governed by the Ministry of Health and Social Affairs (http://www.socialstyrelsen.se/english). One of the authority’s main tasks is to manage healthcare regulation and provide guidelines. As a means of monitoring the healthcare system and to identify best practice, all healthcare providers are mandated to continuously report to the authority’s health data registers. Data are used to establish guidelines and to report on the performance of healthcare providers. In addition, researchers can access information in health data registers after proper ethical approval.

The data set described here includes National Board of Health and Welfare data up to 2013. Depending on the start point of the different health data registries, there is variation in how long back data was available. The Cancer Register provided data from 1958, the National Patient Register from 1964, the In-patient Care Operations Register from 1997, the Cause of Death Register from 1961, the Drug Register from 2005. Table 2 presents an example overview of data obtained from the National Board of Health. This will form the basis for the study of comorbidities and has been helpful in the validation of the data.

**Table 2:** Illustrative example of the data provided by the National Board of Health and Welfare regarding the hip replacement patients

Category	Description
Cancer case	Of the 274,930 total hip replacement patients 79,698 patients had 107,464 cancer cases registered between 1958 and 2013
Death	Of the 274,930 total hip replacements 108,771 patients deceased until 2013
Healthcare events	Of the 274,930 total hip replacements 274,086 underwent inpatient care at 2,910,848 occasions and 249,912 patients underwent outpatient care at 5,131,759 occasions. Additionally between 1997 and 2000 50,827 patients received inpatient care operations. After year 2000 inpatient care operations are registered in inpatient or outpatient care registries. This data base contains a total of 13,573,599 entries of ICD-9 or ICD-10 codes.
Drug usage	The yearly drug usage averaged around 7 million withdrawals by 175,000 patients. The total number of drug withdrawals was 74,427,986.

### Statistics Sweden

Statistics Sweden is an administrative governmental agency with main task to supply statistics for decision-making, debate and research. In addition, they coordinate the Swedish Institute for Official Statistics. Aggregated level data is available for the general public trough the agencies website (www.scb.se). Microdata can be accessed after appropriate ethical approval. For the present project Statistics Sweden contributed with data from three databases under its governance, the Registry for the total population, the longitudinal database for integration studies and the Longitudinal integration database for health insurance and labor market studies (LISA).

From Statistics Sweden we obtained socioeconomic data comprising gender, age, birth location, civil status, number of children, municipality at the time of operation, personal and family income at the time of operation, possible subsidies, health-related leave of absence, and highest level of education at the time of operation among others.

### Data storage and data management

The data from the three main sources are stored on encrypted servers administrated by Center of Registers Västra Götaland. Data from 279,173 primary procedures in 230,424 patients and 16,501 reoperations in 15,842 patients operated from 1992 to 2014 comprise 96 gigabytes in 79 files. For the total hip replacements 279,173 operations on 230,424 patients were successfully matched against the National Board for Health and Welfare and Statistics Sweden data, while for 59 patients the matching failed. This loss in the linkage process equates to 0.025 % of the total number of patients.

For the partial hip replacements 34,781 operations on 33,314 patients were successfully matched against the National Board for Health and Welfare and Statistics Sweden data, while for 1 patients the matching failed (0.003 % of patients). The failures in linkage were in the first part of the process between the SHAR and the National Board of Health and Welfare and were caused by the use of temporary social security numbers (in case of immigration) later changed to definitive numbers, PIN errors and emigration. In the further process no errors could be identified.

The data can be assessed only by SHAR researchers involved in the project. Researchers not employed by SHAR but involved in the project will gain access only to the data needed to the individual study. The data will be structured by statisticians at SHAR. The structured data will then be uploaded to a virtual computer (SODA, Secure On-line Data Access). SODA can be accessed after double identification, individual password and a second temporary password sent to a mobile device of the researchers registered to the service. SODA does not allow users to export or download individual level data and only aggregate level data can be stored on researchers’ own terminals.

The process of data linkage, storage and mangement follows the legal and ethical frameworks as described by the Swedish law and ethical boards. The process and the governance frameworks of register based research have been described by Ludvigsson et al. [17].

## Utility and discussion

The principal goal of this data acquisition is to construct algorithms for the development of a SDM tool intended for Swedish orthopaedic surgeons and their patients in considering THR. Naturally, this data set will be used for other projects as well.

### Shared decision making

One of the main goals is to develop a tool for clinical decision-making in THR. Prior to the present efforts, we have in a series of studies investigated a number of determinants of good and poor outcomes and identified accurate methods to assess those determinants and outcomes [18–28]. We now aim to establish a tool that will provide individualized multidimensional outcome predictions based on information provided by patients (demography, baseline PROMs, and comorbidities) and by clinicians (diagnosis and technical details about appropriate methods and implants). Three main outcome dimensions will be included in the models: patient-reported outcomes (pain, HRQoL, and satisfaction), risk of revision surgery or other reoperation and other adverse events within 30 and 90 days (pneumonia, gastric ulcer, and thromboembolic, cardiovascular, and cerebrovascular complications). In addition, we aim to include expected length of stay, discharge disposition, and return-to-work.

There are several potential benefits of using a decision tool in the assessment of patients for whom THR is considered. First, informing and educating patients about risks and expected outcomes contribute to set realistic expectations. Fulfilling expectations is paramount in achieving patient satisfaction. Second, modifiable risk factors, such as tobacco smoking, distress, and malnutrition, may be identified and improved before surgery. Third, a decision tool may help the orthopaedic surgeon to make individualized recommendations on the ideal implant characteristics such as type of fixation and bearing surface. Fourth, a decision tool may provide information on ideal timing of surgery, if a particular patient’s outcome is expected to be worse if he or she would have surgery later.

It should be emphasized that the decision tool is not meant to replace the surgeons’ assessment of the indication for surgery but to help identifying patients at high risk of poor outcomes and complications, and to facilitate the discussion of timing and expected outcomes. We believe that the identification or confirmation of new and existing factors associated with different outcomes is an important step in improving care with THR. Since individuals electively choose this procedure with the hope of eliminating pain and improving their mobility, it is very likely that some patient will be dissatisfied with their treatment in the absence of such improvements.

### Health economics

From a health economic perspective the linkage between resource use, procedures and quality outcomes is crucial for different types of economic analysis where efficiency and cost-effectiveness are estimated. By making big data from registers available at an individual level analysis based on “real-world evidence” will give additional information and knowledge of evaluation of medical technologies that are not captured in clinical trials. International and national comparison of performance of health care providers is another developing research field where analyses based on open datasets will provide policy-makers as well as clinicians with information about variation and pattern in efficiency. Also evaluation of health care reforms would benefit from benchmarking of intervention effects. The present Swedish reforms introducing freedom of choice and competition across providers raises questions about quality issues and other consequences of the reforms [29]. Access to linked datasets with anonymised data at individual level will give opportunities to multi-level analysis where impact of provider skills and patient case-mix could be separated. This type of analysis is important for both fair benchmarking but also for development of innovative monitoring and reimbursement principles where outcome and value could be considered. One example of analyzing performance of health care providers and evaluate health reforms is the EU-funded EuroHOPE-project which showed considerable variations in volume and outcome both within and across countries [30].

### Reoperation after a primary THR

Reoperation is defined as any further surgical intervention to the hip. If any part(s) or the entire implant are/is removed or exchanged the term revision is used. In Sweden revisions constituted about 80 % of all reoperations during 2013 to 2014. Forty to 50 % of those patients who undergo a reoperation are classified into ASA class III or higher (severe systemic disease or worse physical condition), whereas the corresponding share in primary THR is slightly below 20 %. About 20 % of those patients who are revised for the first time will undergo a second revision and 25 % of those who are revised for a second time will be revised once again. If a revision fails the most common reason for the next one is the same as for the previous one. Thus, frail patients are prone to undergo multiple revision procedures and the results are much inferior compared to primary joint replacement. The purpose of this subproject is to evaluate possible risk factors for revision surgery and especially risk factors associated with multiple revisions.

Identification of risk factors for further intervention(s) when the index primary THR is planned could become a very valuable tool in the decision-making process. Such an instrument could be helpful in avoiding complications if it leads to optimizing the general health of the patient and provides guidelines for choice of surgical method and optimum type of fixation and implant design. This instrument will also provide a basis for an evidence-based discussion with the patient before the decision of performing a primary THR is made.

### Hip fractures

Femoral neck fractures – in particular the displaced ones – are commonly treated with either PHR or THR. In spite of a number of clinical trials, there is yet no clear evidence which type of hip replacement to use for the individual patient. PHR reduces the risk of dislocation but has a particular complication – acetabular erosion – as it articulates towards the cartilage. Since THR includes a plastic cup inserted in the acetabulum erosion is not a problem. However, THRs have in general longer operation time and more bleeding, but if this has any clinical consequences, such as increased mortality is not known. THR may lead to better function and HRQoL, but there is conflicting evidence in randomized studies [31–41]. In particular patient groups, recommendations are rationally based: Frail, inactive individuals with short remaining life span will not benefit from the longer, more complicated THR procedure. Younger individuals without physical limitation pre-fracture will develop acetabular erosion if treated with PHR. But for the majority of “normal” hip fracture patients, clear-cut recommendations are lacking. The uncertainty is illustrated by the variation from 1 to 78 % usage of THR for this patient cohort in different Swedish hospitals [33].

The data will allow us to compare PHR and THR as treatment for femoral neck fractures, in terms of reoperation and mortality, and to make clinical recommendations on which implant to use in different major subgroups of fracture patients. In addition, the true incidence and risk factors of particular complications such as dislocation can be deduced. Furthermore, any benefits of specific implant designs can be analyzed. By linking QR data with the health data register of the National Board of Health and Welfare and Statistics Sweden, several otherwise unknown confounding factors are added to the analyses. Thus, this data set will reflect the complex clinical setting better, when more details are known about the included patients.

## Conclusions

In this paper we outlined the data acquisition process and what purpose this data will serve. The data acquisition process outlined here is only possible because of the existence of the 10-digit PIN applied at all levels of the administrative and healthcare system. The existence of the 10-digit PIN in a population largely supportive of these types of analyses offers a unique opportunity for researchers. This model, of interrogation is, however, under threat. At the moment, QRs do not require written consent from the patients. Law changes at the European Union level might require written consent. Gathering written consent at the level of ten thousands imposes an administrative burden few registries can afford. Currently, registries function on the ‘opt-out’ principle. Patients can require to see their data and they can withdraw at any time. During the 35 years history of SHAR so far only five patients wished to see their data and of them two decided to opt-out.

Protection of medical data and individual privacy is top priority. As we have outlined here Sweden offers a working model that protects individual patients but at the same time booster innovation and research that have the potential to improve care and support value-based healthcare.

## Abbreviations

ASA: American Society of Anaesthesiologists
EU: European Union
HRQoL: Health Related Quality of Life
LISA: Longitudinal integration database for health insurance and labor market studies
PHR: Partial Hip Replacement/hemi-arthroplasty
PIN: Personal identity number
PROM: Patient-reported outcome measurement
QR: Swedish Healthcare Quality Registers
SDM: Shared decision-making
SHAR: Swedish Hip Arthroplasty Register
SODA: Secure on-line data access
THR: Total hip replacement
